# Semi-open rhinoplasty: a new Maxillofacial technique

**DOI:** 10.1186/1746-160X-8-13

**Published:** 2012-04-30

**Authors:** Francesco Inchingolo, Marco Tatullo, Massimo Marrelli, Alessio D Inchingolo, Roberto Corelli, Angelo M Inchingolo, Paolo Flace, Raffaele Cagiano, Gianna Dipalma, Fabio M Abenavoli

**Affiliations:** 1 Department of Dental Sciences and Surgery, University of Bari, Bari, Italy; 2 Department of Basic Medical Sciences, University of Bari, Bari, Italy; 3 Department of “Head and Neck Diseases”, Hospital “Fatebenefratelli”, Rome, Italy; 4 Unit of Maxillofacial Surgery, Calabrodental clinic, Crotone, Italy; 5 Dental School, University of Bari, Bari, Italy; 6 Department of Maxillofacial Surgery, General Hospital, Bari, Italy; 7 Department of Surgical, Reconstructive and Diagnostic Sciences, General Hospital, Milan, Italy; 8 Tecnologica Research Institute, Biomedical section, Crotone, Italy; 9 Department of Biomedical Sciences and Human Oncology, Medical Faculty, University of Bari, Bari, Italy; 10 Department Dental Sciences and Surgery, Piazza Giulio Cesare – Policlinico, 70124, Bari, Italy

**Keywords:** Rhinoplasty, Maxillofacial technique, Plastic surgery

## Abstract

**Background:**

Rhinoplasty "open" represents a surgical technique to access to the internal structures of the nose; it is an alternative to more traditional "closed" rhinoplasty. However, both these techniques have some advantages and some disadvantages. In this work the authors describe a case that shows the steps of a new surgical technique: the “semi-open” rhinoplasty.

**Methods:**

The "semi-open" technique is performed by making an incision to access on the mucosa of both the nostrils, and through this access we separate the cartilages of the columella from the alar cartilages, debriding them at the domus. With such access we can perform any type of rhinoplasty surgery with functional or aesthetic purposes.

**Discussions:**

Traditional techniques have undoubtedly some advantages and some disadvantages. The "semi-open" technique has the several advantages of the open technique, and it does not involve the presence of post-surgical scars.

**Conclusions:**

This innovative technique provides great predictability and minimal postoperative discomfort, with no aesthetic damage.

## Introduction

Rhinoplasty "open" is a surgical technique that allows, through the transverse incision of the columella [1], to access to the osteo-cartilaginous structures of the nose and to make all appropriate changes course to achieve functional and aesthetic purposes, thanks to a direct and wide vision of surgical site. The technique "open" represents a surgical mode to access to the internal structures of the nose, an alternative to more traditional "closed" rhinoplasty, which is realized through incisions made inside the nostrils, in correspondence with the area to be treated.

Both these techniques have certainly some advantages and some disadvantages [2], therefore, the authors wanted to test a new technique that shows the advantages of "open" and "closed" procedure: this new technique is called "semi-open", and in this work the authors describe a case that shows the steps of this surgical technique.

## Materials and methods

**Figure 1:**
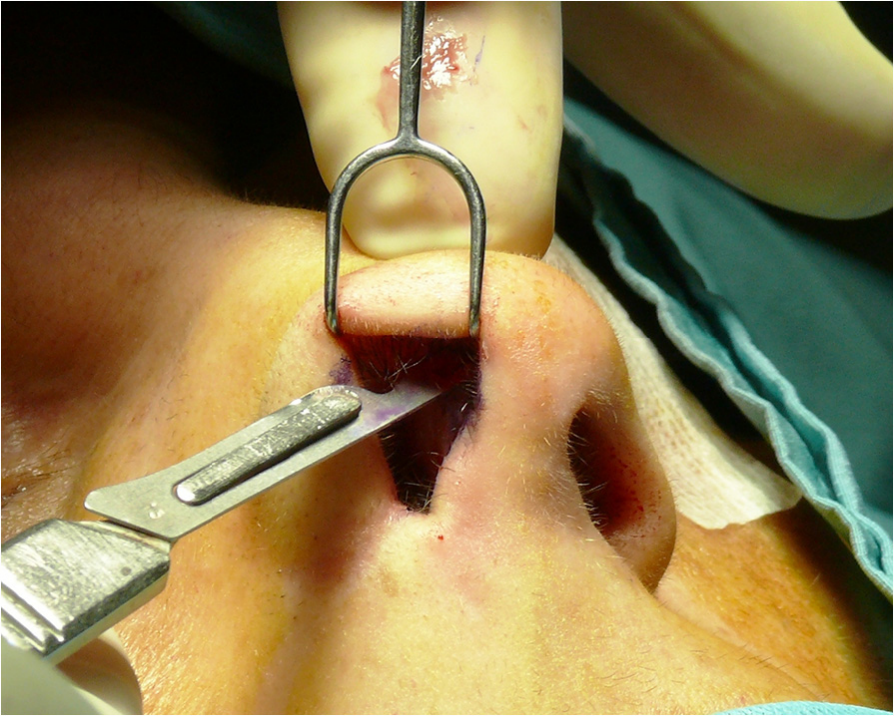
The incision to access on the mucosa of the nostril.

**Figure 2:**
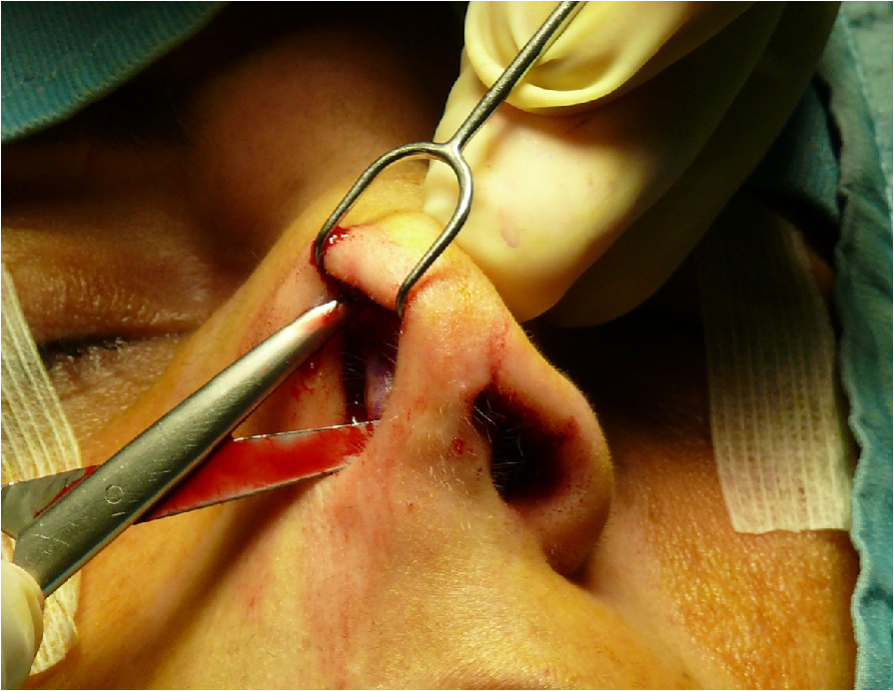
Separation of the cartilages of the columella from the alar cartilages.

**Figure 3:**
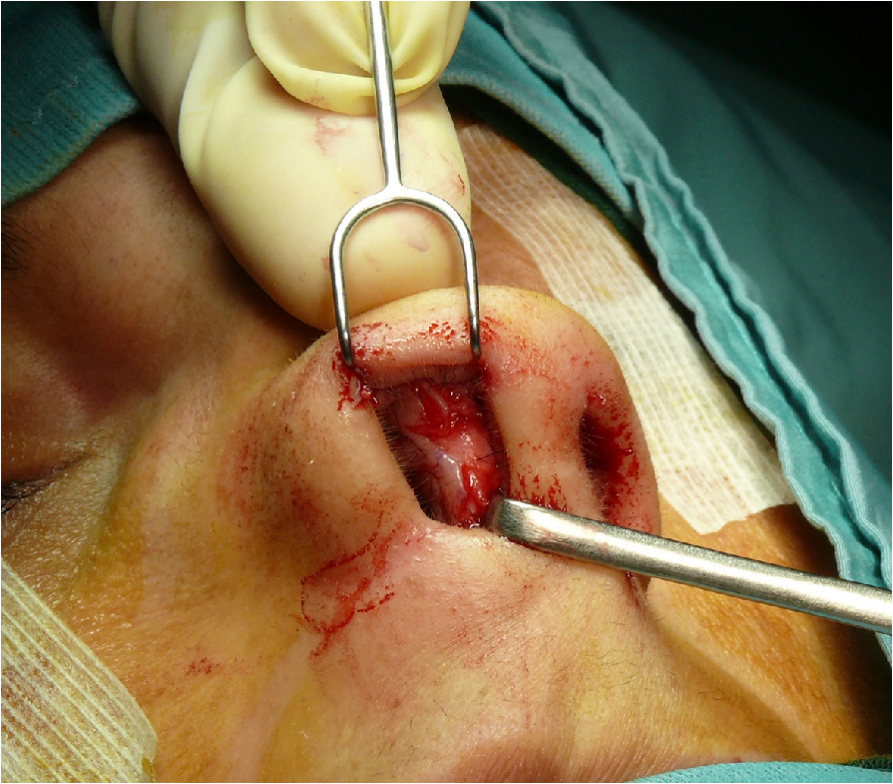
The alar cartilages exposed.

**Figure 4:**
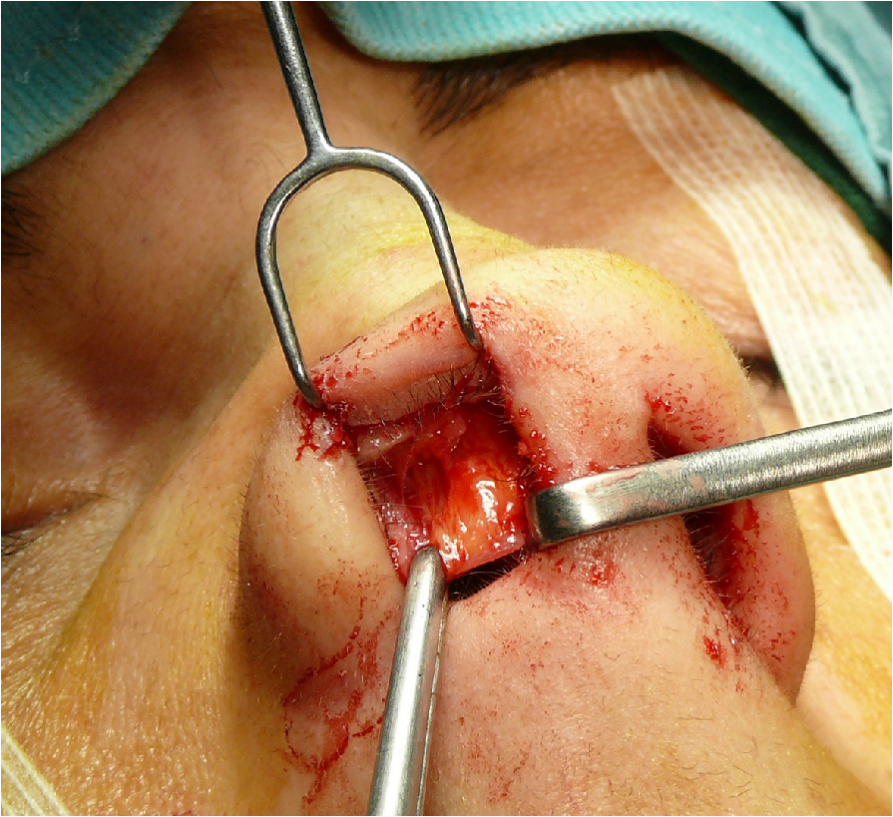
An intraoperative procedure for withdraw cartilage.

**Figure 5:**
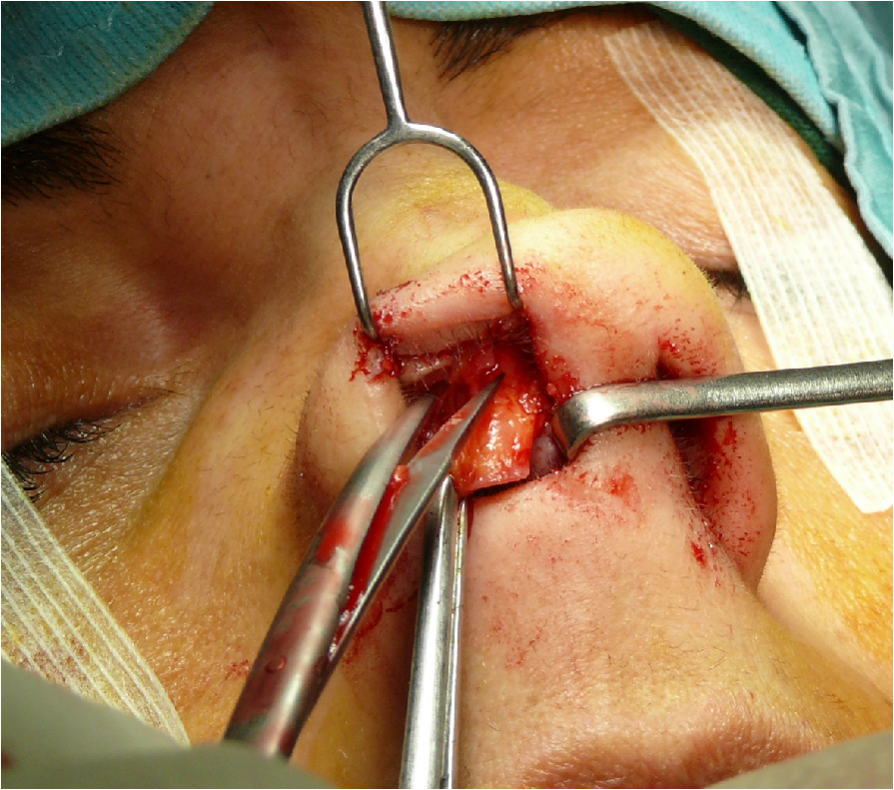
An intraoperative procedure for withdraw cartilage.

**Figure 6:**
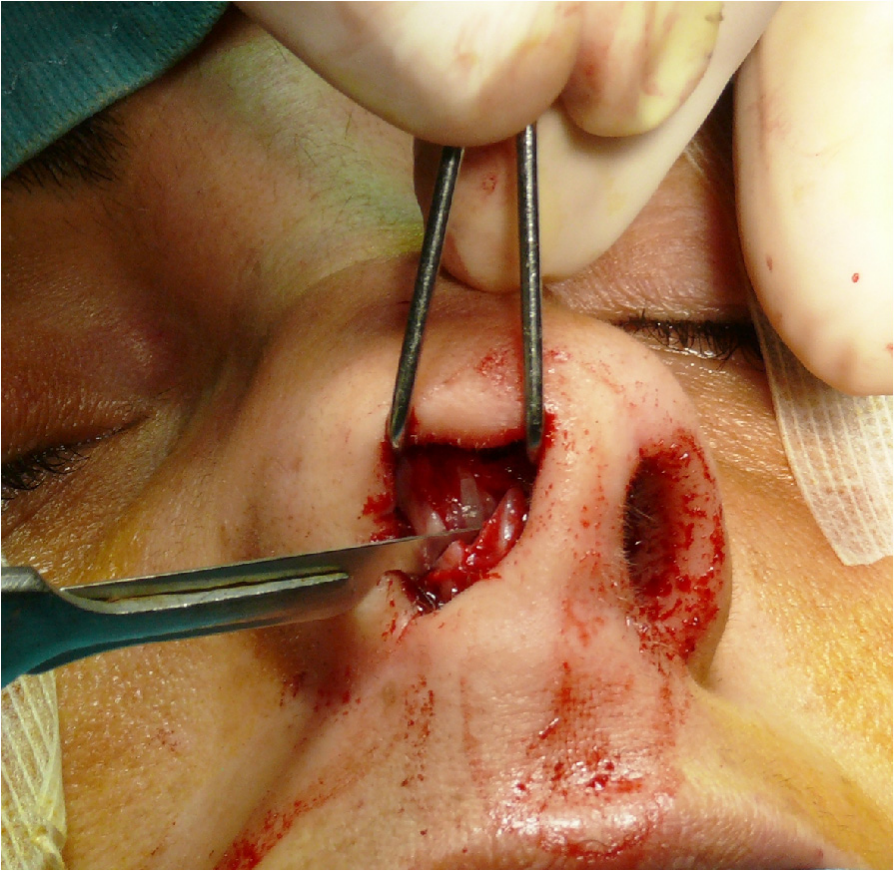
An intraoperative procedure for withdraw cartilage.

**Figure 7:**
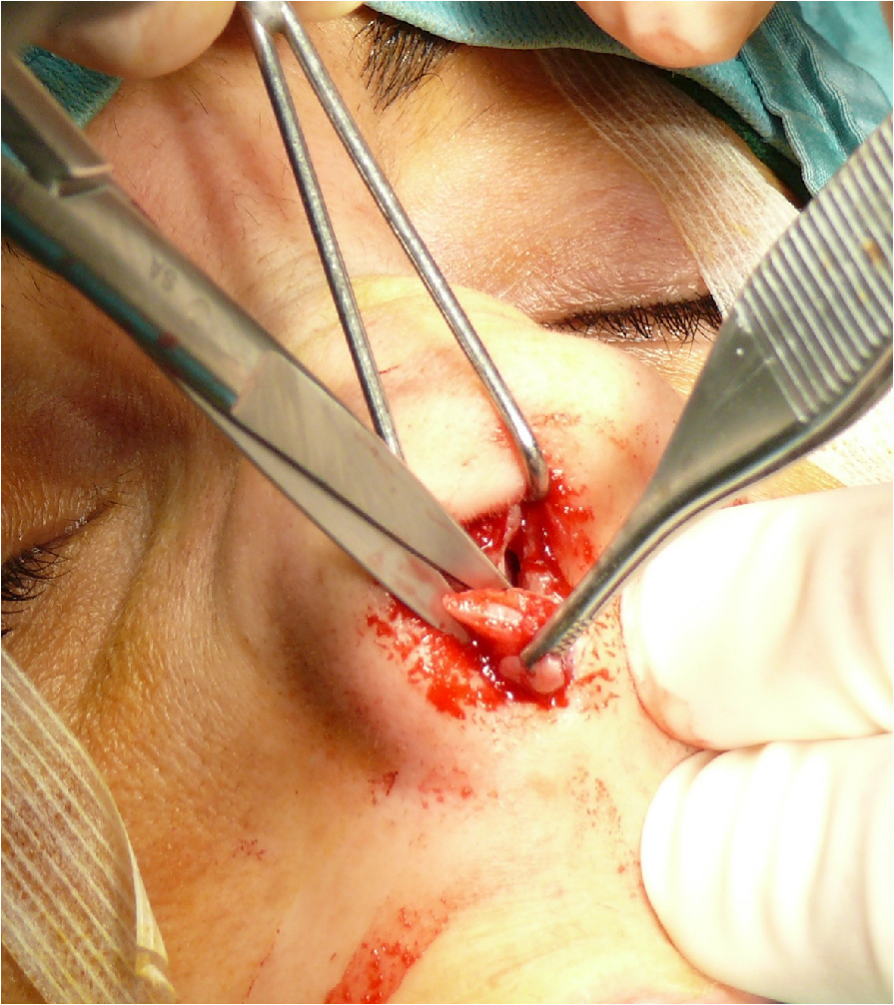
An intraoperative procedure for withdraw cartilage.

The "semi-open" technique is performed by making an incision to access on the mucosa of the nostril (Figure 1), and through this access we separate the cartilages of the columella from the alar cartilages (Figure 2), the same operation is performed in the contralateral nostril, debriding them at the domus. Later, with the alar cartilages totally exposed (Figure 3) we can perform any type of rhinoplasty surgery with functional or aesthetic purposes. With such access is also possible to completely detach the nasal septum, in order to then perform the preferred technique for correcting a deviation, or withdraw cartilage (Figures 4, 5, 6 and 7) .

**Figure 8:**
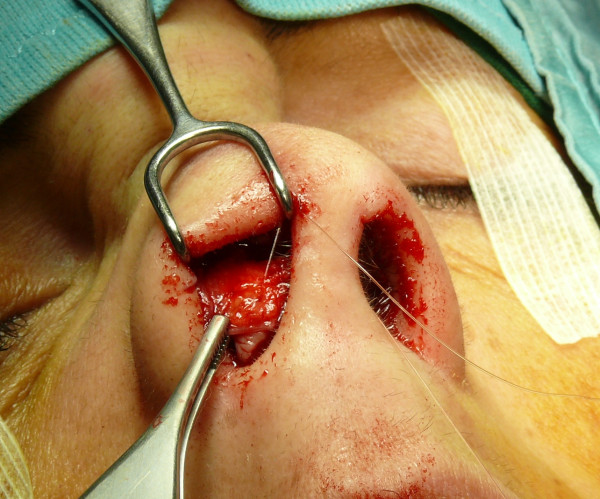
The suture is performed firstly with separate stitches in the submucosa and then on the mucosa.

**Figure 9:**
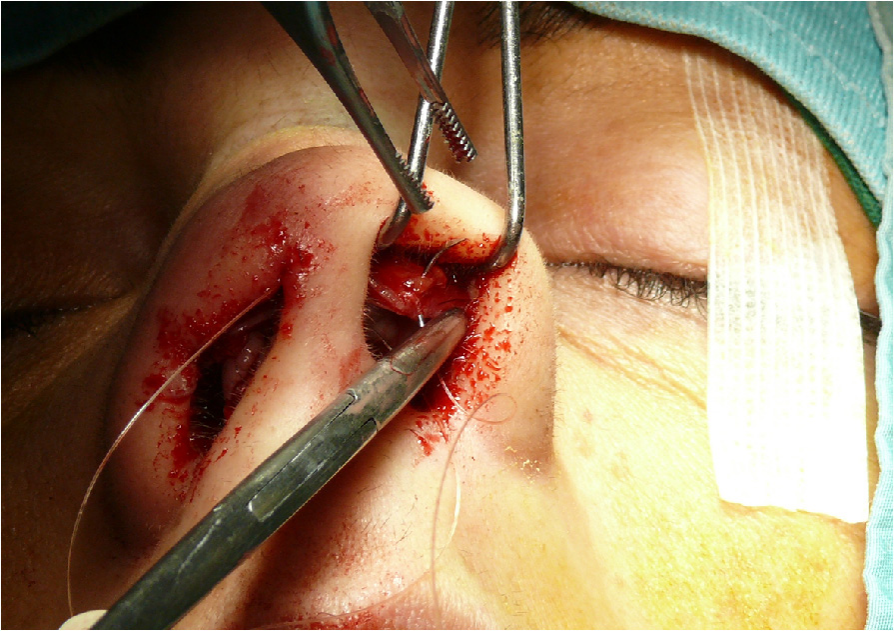
The suture is performed firstly with separate stitches in the submucosa and then on the mucosa.

**Figure 10:**
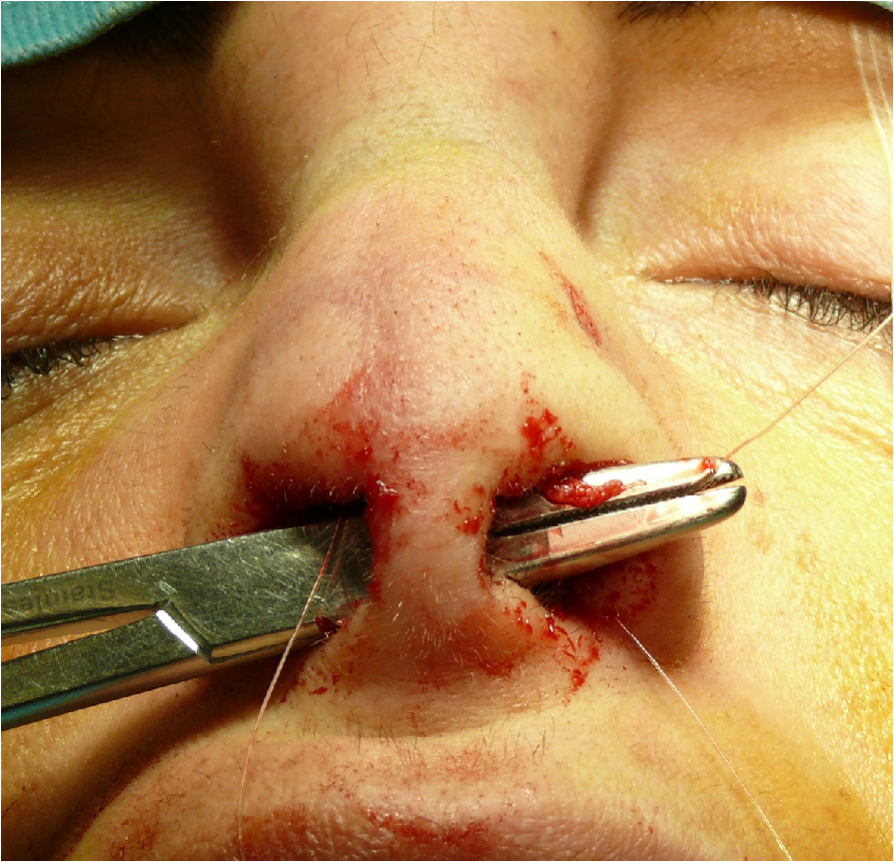
The suture is performed firstly with separate stitches in the submucosa and then on the mucosa.

**Figure 11:**
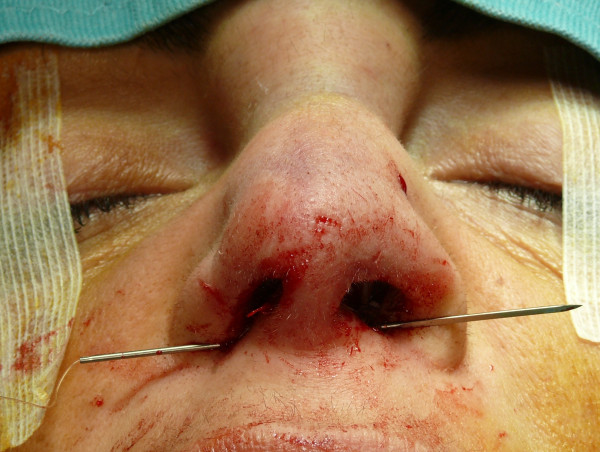
The suture is performed firstly with separate stitches in the submucosa and then on the mucosa.

**Figure 12:**
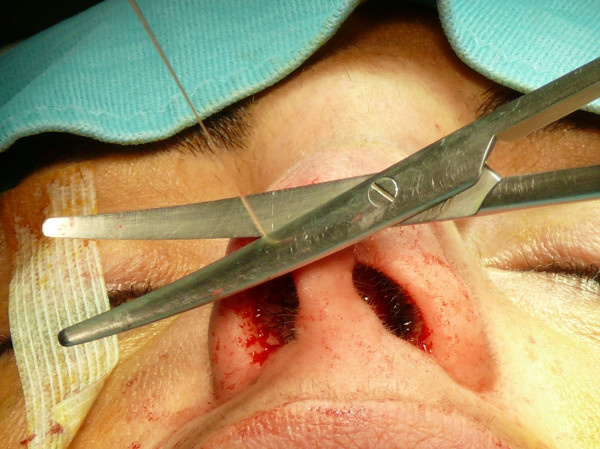
The suture is performed firstly with separate stitches in the submucosa and then on the mucosa.

**Figure 13:**
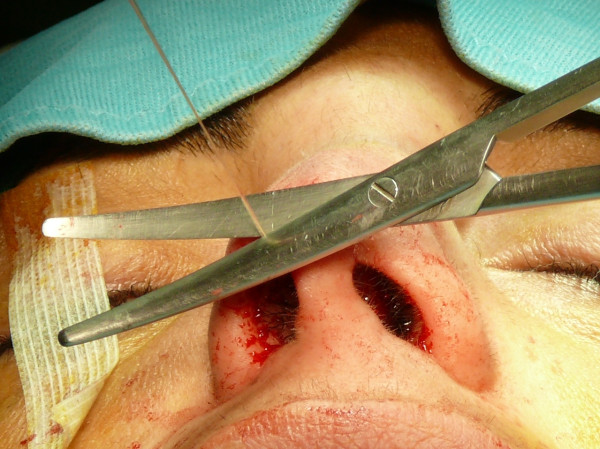
The suture is performed firstly with separate stitches in the submucosa and then on the mucosa.

The suture is done with separate stitches in the submucosa and mucosa (Figures 8, 9, 10, 11, 12 and 13).

The intervention lasts absolutely equivalent to the duration of the traditional techniques [3].

During The post-operatory period, the authors have treated the patients with local application of an antiphlogistic ointment and with the administration of bromeline pills in order to reduce the swelling [4].

## Discussion

Traditional techniques have undoubtedly some advantages and some disadvantages [5]. The advantages of the "open" rhinoplasty are, for example, a greater intra-operative visibility, high precision in performing corrective action, symmetrical on both sides of the nose; in fact, by using the approach of "open" rhinoplasty, we can make any change under direct vision. In addition, this technique creates a large surgical access that makes it possible to model the shape of the nose by inserting and fixing cartilage grafts; the “open” rhinoplasty makes it more easy and accurate not only the removal of cartilage from the septum, but also more accurate and stable the placement of the grafts in the different sites [6].

Despite the numerous advantages, there are also some disadvantages in the open rhinoplasty, such as, for example, a post-operative course longer and a greater presence of edema on the region of the columella, frequently accompanied with paranasal hematomas; another poorly aesthetic result is the presence of a transverse scar in correspondence of the columella [7].

The literature of the last 15 years has highlighted that many surgeons prefer the approach of "open" rhinoplasty for the greater facility in performing complex interventions on the osteo-cartilaginous tissues of the nose and for ever greater predictability of results, although the closed technique allows to achieve a minor trauma for the soft tissues with good aesthetic conditions in the post-operative course: the "semi-open" technique has the several advantages of the open technique, and it does not involve the presence of post-surgical scars.

## Conclusions

The "semi-open" technique allows operating times comparable to the traditional techniques, in addition, it allows to have an intra-operative visual field very wide, equivalent to that which can be achieved using the open technique, but without leaving any external scar; this innovative technique provides great predictability and minimal postoperative discomfort, with no aesthetic damage.

## Competing interests

The authors declare that they have no competing interests.

## Authors' contributions

FI: participated in the surgical treatment and in the follow-up of this patient MT: participated in the design of this technique, drafted the manuscript and reviewed the literature sources FMA: participated in the surgical treatment and in the follow-up of this patient MM: participated in the design of this technique and in the follow-up of this patient ADI and PF: revised the literature sources RC and RC: participated in the surgical treatment and in the follow-up of this patient AMI: documented this case report with digital pictures GD: participated in the follow-up of patients All the authors read and approved the final manuscript.

## Authors’ information

Written informed consent was obtained from the patient for publication of this case report and accompanying images. A copy of the written consent is available for review by the Editor-in-Chief of this journal.

## References

[B1] Rohrich RJ Hoxworth RE Kurkjian TJ The role of the columellar strut in rhinoplasty: indications and rationale Plast Reconstr Surg 2012 129 1 118e 125e 10.1097/PRS.0b013e3182362b7a 22186526

[B2] DeFatta RJ Ducic Y Adelson RT Sabatini PR Comparison of closed reduction alone versus primary open repair of acute nasoseptal fractures J Otolaryngol Head Neck Surg 2008 37 4 502 506 19128583

[B3] Ponsky D Eshraghi Y Guyuron B The frequency of surgical maneuvers during open rhinoplasty Plast Reconstr Surg 2010 126 1 240 244 10.1097/PRS.0b013e3181dc54da 20595871

[B4] Inchingolo F Tatullo M Marrelli M Inchingolo AM Picciariello V Inchingolo AD Dipalma G Vermesan D Cagiano R Clinical trial with bromelain in third molar exodontia Eur Rev Med Pharmacol Sci 2010 14 9 771 774 21061836

[B5] Berghaus A Rhinoplasty: open or closed technique? HNO 2010 58 9 878 881 10.1007/s00106-009-2041-x 20668827

[B6] Quatela VC Jacono AA Structural grafting in rhinoplasty Facial Plast Surg 2002 18 4 223 232 10.1055/s-2002-36490 12524594

[B7] Han SK Woo HS Kim WK Extended incision in open-approach rhinoplasty for asians Plast Reconstr Surg 2002 109 6 2087 2096 10.1097/00006534-200205000-00047 11994619

